# Supplementary material for: Early genetic evolution of driver mutations in uveal melanoma
Source: Nat Commun. 2025 Dec 12;16:11322. doi: 10.1038/s41467-025-66428-x (PMC12722419; doi:10.1038/s41467-025-66428-x)
Supplement: Supplementary file 1 — Supplementary Information [file 41467_2025_66428_MOESM1_ESM.pdf]

SUPPLEMENTARY INFORMATION

Supplementary Table 1. Summary of clinical and demographic features of all study subjects (n=1140 cases).

Features	Values
Age at study entry, years	
Median (range)	64.3 (18-99)
Mean (SD)	62.5 (13.63)
Male sex, No. (%)	590 (51.75)
Eye, Right, No. (%)	578 (50.7%)
Iris color, No. (%)	
Blue/green	453 (39.7%)
Brown	167 (14.7%)
Intermediate	89 (7.8%)
Not specified	431 (37.8%)
Ciliary body involvement, No. (%)	201 (17.6%)
Tumor diameter, mm	
Median (range)	12.1 (3-29)
Mean (SD)	12.6 (+/-3.9)
Tumor thickness, mm	
Median (range)	4.5 (1-18)
Mean (SD)	5.5 (+/-3.1)
15-GEP, No. (%)	
Class 1	716 (62.8%)
Class 2	424 (37.2%)
PRAME, No. (%)	
Negative (–)	757 (66.4%)
Positive (+)	383 (33.6%)
Death, No. (%)	241 (21.1%)
Time to death or last follow-up, months	
Median (range)	52.8 (0-101.7)
Mean (SD)	52.4 (+/- 22.1)
Metastasis, No. (%)	229 (20.1%)
Time to metastasis or last follow-up, months	
Median (range)	50.8 (0-101.7)
Mean (SD)	49.1 (+/- 24.0)
Local recurrence, No. (%)	54 (4.7%)
Time to local recurrence, months	
Median (range)	28.5 (3.5-82.2)
Mean (SD)	30.2 (18.6)
Metastasis after local recurrence, No. (%)	28 (51.9%)

Abbreviations: 15-GEP, 15-gene expression profile; SD, standard deviation; No., number.

Supplementary Table 2. Univariate Cox regression analyses of metastasis-free survival and overall survival (n=1133 cases).*

Risk Factor	Metastasis-Free Survival		Overall Survival	
	**Univariate HR (95% CI)**	***P***	**Univariate HR (95% CI)**	***P***
15-GEP Class 2	11.0 (7.8,15.5)	2.3×10^{-43}	6.3 (4.7,8.5)	3.1×10^{-35}
PRAME(+)	3.3 (2.5,4.2)	2.2×10^{-18}	2.9 (2.2,3.7)	6.2×10^{-16}
GNAQ	0.8 (0.6,1.0)	0.05	0.7 (0.5,0.9)	0.001
GNA11	1.1 (0.9,1.5)	0.4	1.4 (1.1,1.9)	0.006
PLCB4	1.5 (0.7,3.3)	0.3	1.2 (0.5,2.6)	0.7
CYSLTR2	2.0 (0.8,4.9)	0.1	0.7 (0.2,2.9)	0.6
BAP1	5.9 (4.5,7.8)	1.0×10^{-26}	4.3 (3.3,5.6)	2.5×10^{-27}
SF3B1	0.5 (0.3,0.7)	0.0009	0.5 (0.3,0.7)	0.0005
EIF1AX	0.2 (0.1,0.3)	4.8×10^{-10}	0.4 (0.3,0.6)	2.1×10^{-6}
CCF_{BAP1}**	1.0 (1.0,1.0)	1.0	1.0 (1.0,1.0)	0.8
CCF_{SF3B1}**	1.03 (1.0,1.1)	0.3	1.0 (1.0,1.1)	0.8
CCF_{EIF1AX}**	1.00 (1.0,1.0)	0.8	0.99 (1.0,1.0)	0.1

*Excludes 7 cases with metastasis at baseline.

**Analysis of CCF_{BAP1}, CCF_{SF3B1}, and CCF_{EIF1AX} only included cases with a *BAP1* mutation (and copy number call for the *BAP1* locus) (n = 283 cases), *SF3B1* mutation (n = 189 cases), or *EIF1AX* mutation (n = 295 cases), respectively.

Abbreviations: 15-GEP, 15-gene expression profile; (+), positive; HR, hazard ratio; CI, confidence interval; CCF, cancer-cell fraction; *GNAQ*, *GNA11*, *PLCB4*, *CYSLTR2*, *BAP1*, *SF3B1* and *EIF1AX* indicate the genes pathogenic mutations in these.

Supplementary Table 3. Multivariate Cox regression survival analysis (n=1133 cases).*

Risk Factor Pairs	Metastasis-Free Survival		Overall Survival	
	**Multivariate HR (95% CI)**	***P***	**Multivariate HR (95% CI)**	***P***
15-GEP Class 2	10.1 (6.5,15.7)	$1.2x10^{-24}$	5.7 (3.8,8.6)	$2.6x10^{-16}$
BAP1	1.1 (0.8,1.6)	0.5	1.2 (0.8,1.7)	0.5
15-GEP Class 2	12.8 (8.8,18.6)	$3.6x10^{-40}$	6.4 (4.7,8.8)	$1.5x10^{-31}$
SF3B1	1.7 (1.1,2.8)	0.03	1.1 (0.7,1.7)	0.8
15-GEP Class 2	9.5 (6.6,13.8)	$3.1x10^{-33}$	6.5 (4.6,9.0)	$1.1x10^{-28}$
EIF1AX	0.6 (0.4,1.1)	0.08	1.1 (0.7,1.6)	0.8
15-GEP Class 2	11.2 (7.9,15.7)	$3.7x10^{-43}$	6.2 (4.6,8.3)	$1.2x10^{-33}$
GNAQ	1.1 (0.8,1.4)	0.5	0.9 (0.7,1.1)	0.3
15-GEP Class 2	11.2 (7.9,15.7)	$1.7x10^{-43}$	6.2 (4.6,8.3)	$3.2x10^{-34}$
GNA11	0.9 (0.7,1.2)	0.4	1.2 (0.9,1.5)	0.2
BAP1	5.9 (4.4,7.8)	$7.3x10^{-35}$	4.2 (3.2, 5.5)	$7.9x10^{-26}$
GNAQ	1.0 (0.7,1.2)	0.7	0.8 (0.6,1.0)	0.05
BAP1	5.9 (4.5,7.8)	$2.1x10^{-35}$	4.2 (3.3, 5.5)	$1.5x10^{-26}$
GNA11	1.0 (0.8,1.3)	0.9	1.3 (1.0,1.7)	0.05
PRAME(+)**	6.7 (3.2,14.2)	$6.2x10^{-7}$	2.7 (1.5,5.0)	0.001
SF3B1**	0.8 (0.4,1.6)	0.5	0.8 (0.4,1.4)	0.4

* Excludes 7 cases with metastasis at baseline.

**Only Class 1 cases included in this subanalysis (n=715 cases).

Abbreviations: 15-GEP, 15-gene expression profile; (-), (+), positive; HR, hazard ratio; CI, confidence interval; *GNAQ*, *GNA11*, *BAP1*, *SF3B1* and *EIF1AX* indicate pathogenic mutations in these genes. *PLCB4* and *CYSLTR2* not included due to small number of cases.

Supplementary Table 4. Comparison of clinical and molecular features associated with cancer cell fraction. Table providing the statistical analysis of discrete variables associated with cancer cell fraction for patients with *BAP1* mutations and quality copy number calls (n=287 cases), *SF3B1* (n=190 cases), and *EIF1AX* mutations (n=295 cases). Variables were analyzed by two-tailed Wilcoxon rank-sum test.

Dichotomous Variable		CCF	CCF		
**Group 1**	**Group 2**		**Group 1***	**Group 2***	***P*-value**
Small Tumors	Larger Tumors	BAP1	87.8 ± 3.8	84.9 ± 1.1	0.3
		SF3B1	83.0 ± 4.4	94.3 ± 0.9	0.002
		EIF1AX	89.9 ± 3.2	95.1 ± 0.8	0.04
Class 1	Class 2	BAP1	79.6 ± 4.9	85.4 ± 1.1	0.2
		SF3B1	93.1 ± 0.9	96.5 ± 1.2	0.9
		EIF1AX	94.4 ± 0.9	94.7 ± 2.1	0.2
PRAME(-)	PRAME(+)	BAP1	85.5 ± 1.3	84.6 ± 1.8	0.9
		SF3B1	91.4 ± 2.0	94.1 ± 0.9	0.5
		EIF1AX	94.4 ± 0.9	94.9 ± 1.5	0.09
No CB Involvement	CB Involvement	BAP1	85.3 ± 1.2	84.4 ± 2.5	0.8
		SF3B1	92.5 ± 1.1	97.0 ± 0.6	0.8
		EIF1AX	94.8 ± 0.8	90.5 ± 3.9	0.04

* Mean ± Standard Error

Abbreviations: CCF, Cancer cell fraction; (-), negative; (+), positive; CB, ciliary body.

Supplementary Table 5. Univariate Cox regression analyses of metastasis-free survival and overall survival in Class 1 (n=467 cases) and Class 2 (n=278 cases) tumors without metastasis at baseline.

Mutated Gene	15-GEP Class	Metastasis-Free Survival		Overall Survival	
		Hazard Ratio (95% CI)	*P*	Hazard Ratio (95% CI)	*P*
*BAP1**	Both Classes	1.0 (1.0, 1.0)	1.0	1.0 (1.0, 1.0)	0.8
	Class 1	1.0 (0.9, 1.0)	0.5	1.0 (0.9,1.0)	0.5
	Class 2	1.0 (1.0, 1.0)	0.8	1.0 (1.0,1.0)	0.7
*SF3B***	Both Classes	1.0 (1.0, 1.1)	0.3	1.0 (1.0, 1.1)	0.3
	Class 1	1.0 (1.0, 1.1)	0.3	1.0 (1.0, 1.1)	0.3
	Class 2	0.9 (0.6, 1.2)	0.4	0.5 (0.04, 6.0)	0.6
*EIF1AX***	Both Classes	1.0 (1.0, 1.0)	0.8	1.0 (1.0, 1.0)	0.1
	Class 1	1.0 (1.0, 1.0)	1.0	1.0 (1.0, 1.0)	0.1
	Class 2	1.0 (0.9, 1.1)	0.7	1.0 (1.0, 1.1)	0.8

* Indicates samples with a *BAP1* mutation and evaluable chromosome 3 copy number call (confidence score 2 or 3) (n=283 cases).

** Indicates samples with a respective *SF3B1* (n=189 cases) or *EIF1AX* (n=295 cases) mutation.

Abbreviations: 15-GEP, 15-gene expression profile; MFS, metastasis-free survival; OS, overall survival; CI, confidence interval.

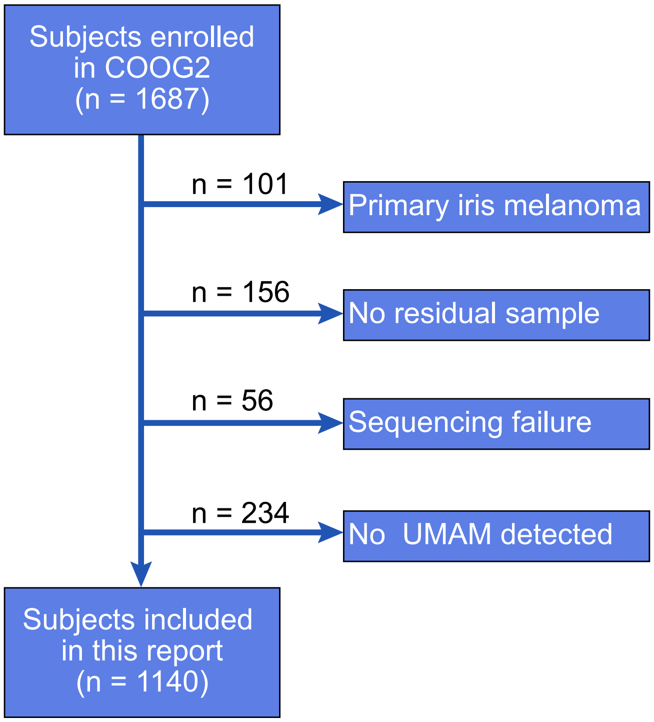

Supplementary Figure 1. Cohort Summary for COOG2.2 Study. Overview of subjects included in Collaborative Ocular Oncology Group Study 2 Report Number 2 (COOG2.2). Abbreviations: Uveal melanoma-associated mutation.

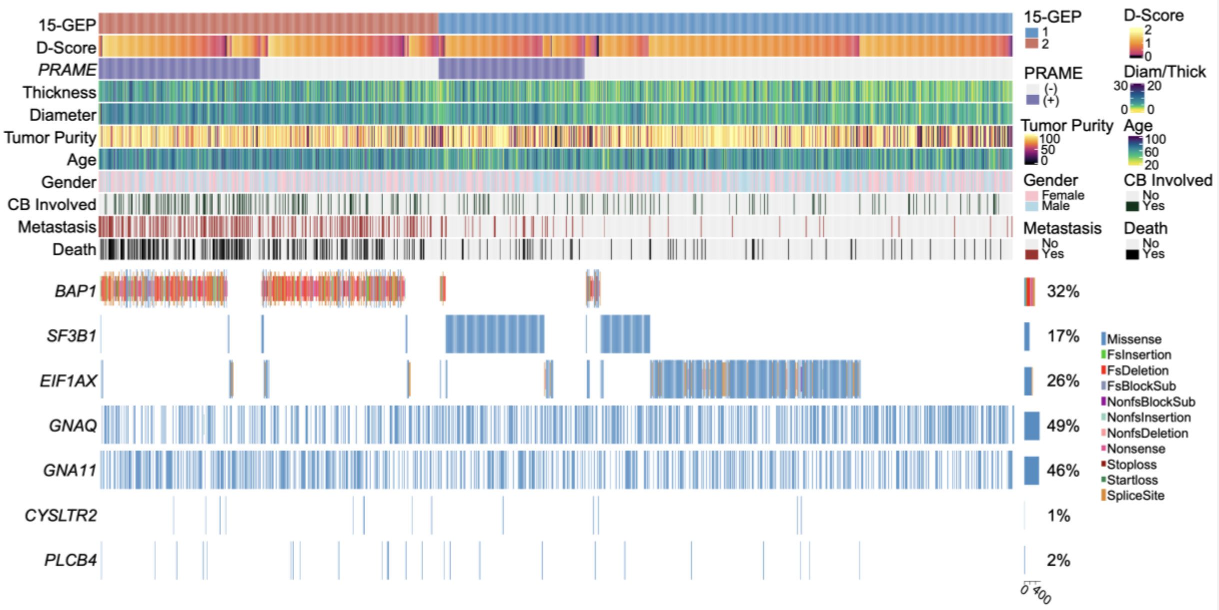

Supplementary Figure 2. Oncoprint Summarizing Cohort by 15-GEP Class and PRAME Status. Oncoprint for 1140 primary uveal melanomas with equivalent data as Figure 1, with samples sorted according to 15-GEP Class and PRAME status followed by *BAP1*, *SF3B1*, and *EIF1AX* mutation and decreasing discriminant score to emphasize the genetic landscape associated with 15-GEP Class and *PRAME* status. Variant types described in Methods. Relevant data provided in Source Data file.

Abbreviations: 15-GEP, 15-gene expression profile; (-), negative; (+), positive; Diam, tumor diameter (mm); Thick, tumor thickness (mm); mm, millimeter; CB, ciliary body; D-score, 15-GEP support vector machine discriminant score; Fs, Frameshift; Nonfs, Non-frameshift; Sub, substitution.

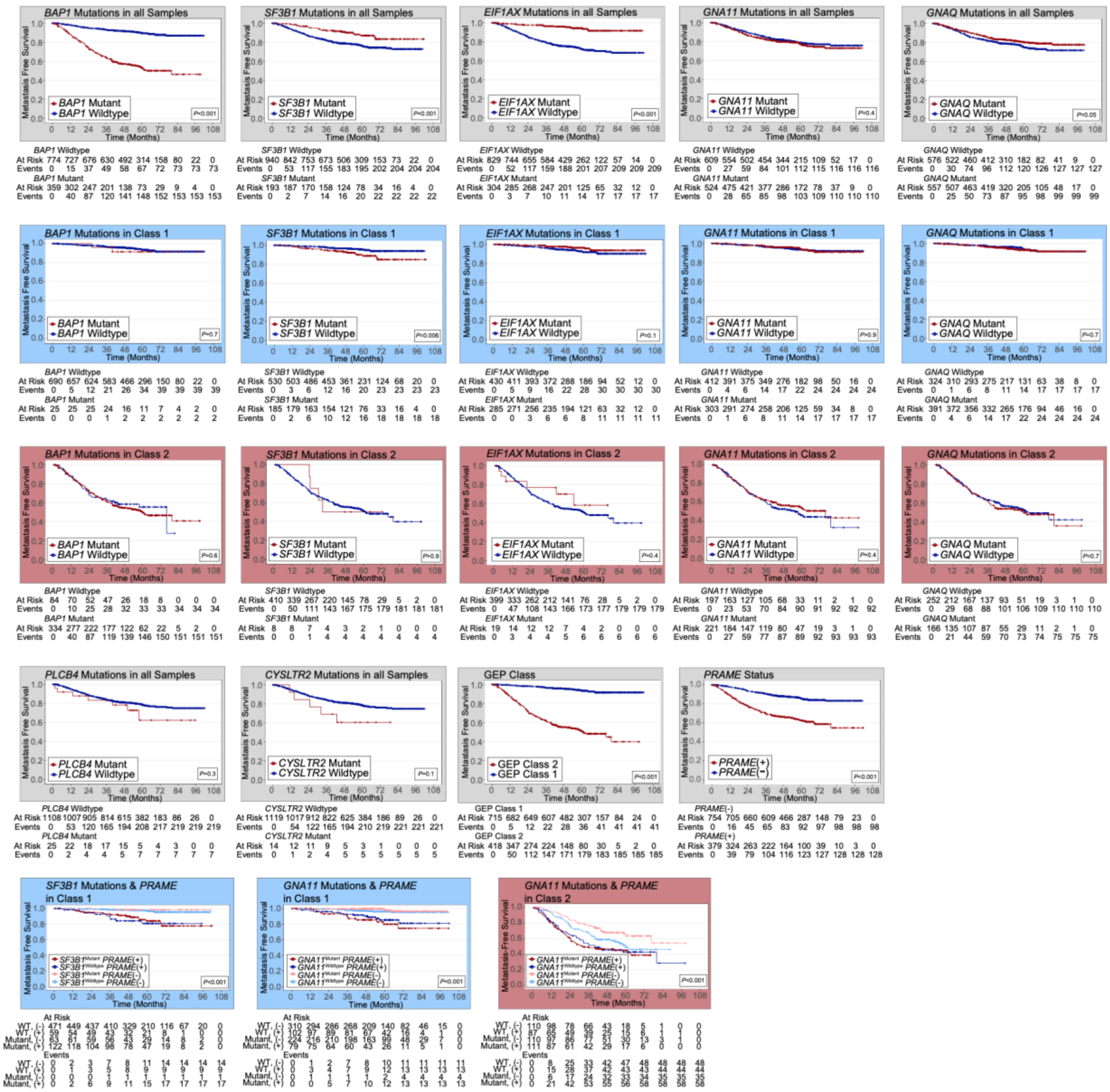

Supplementary Figure 3. Metastasis-Free Survival by Mutation Status. Kaplan-Meier survival curves for metastasis-free survival in patients with detectable UMAMs in all samples (gray outline box) (n=1133 cases), class 1 (blue outline box) (n=715 cases), and class 2 (red outline box) (n=418 cases). The last row depicts survival curves for *SF3B1* mutations or *GNA11* mutations stratified by *PRAME* status in Class 1 and Class 2 UM. Data including exact p-values are available in the Source Data file.

Abbreviations: 15-GEP, 15-gene expression profile; (-), negative; (+), positive; MFS, metastasis-free survival; WT, wildtype.

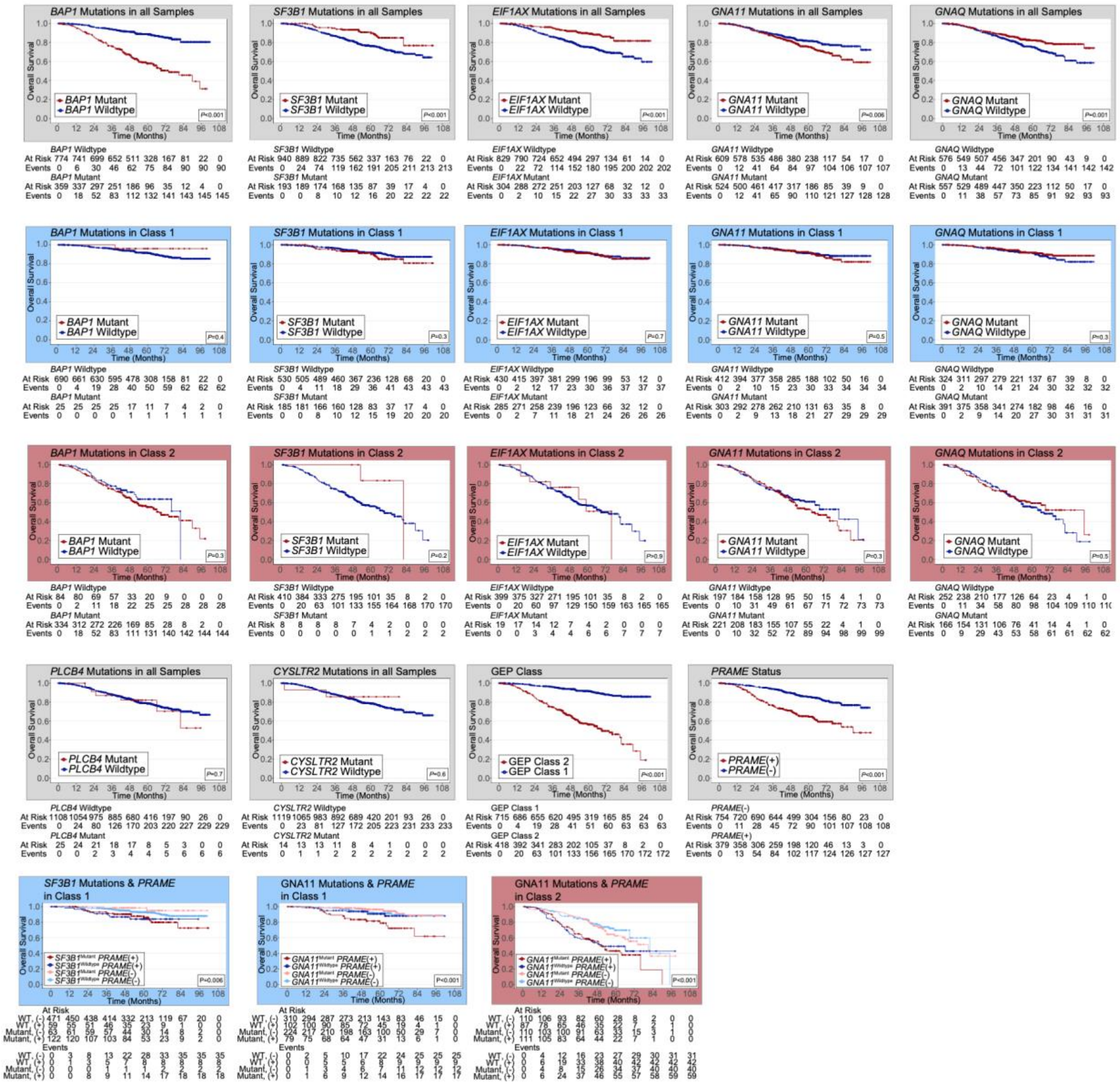

Supplementary Figure 4. Overall Survival by Mutation Status. Kaplan-Meier survival curves for overall survival in patients with detectable uveal melanoma-associated mutations in all samples (gray outline box) (n=1133 cases), Class 1 (blue outline box) (n=715 cases), and Class 2 (red outline box) (n=418 cases). The last row depicts survival curves for *SF3B1* mutations or *GNA11* mutations stratified by *PRAME* status in Class 1 and Class 2 UM. Data including exact p-values are available in the Source Data file.

Abbreviations: 15-GEP, 15-gene expression profile; (-), negative; (+), positive; OS, overall survival; WT, wildtype.

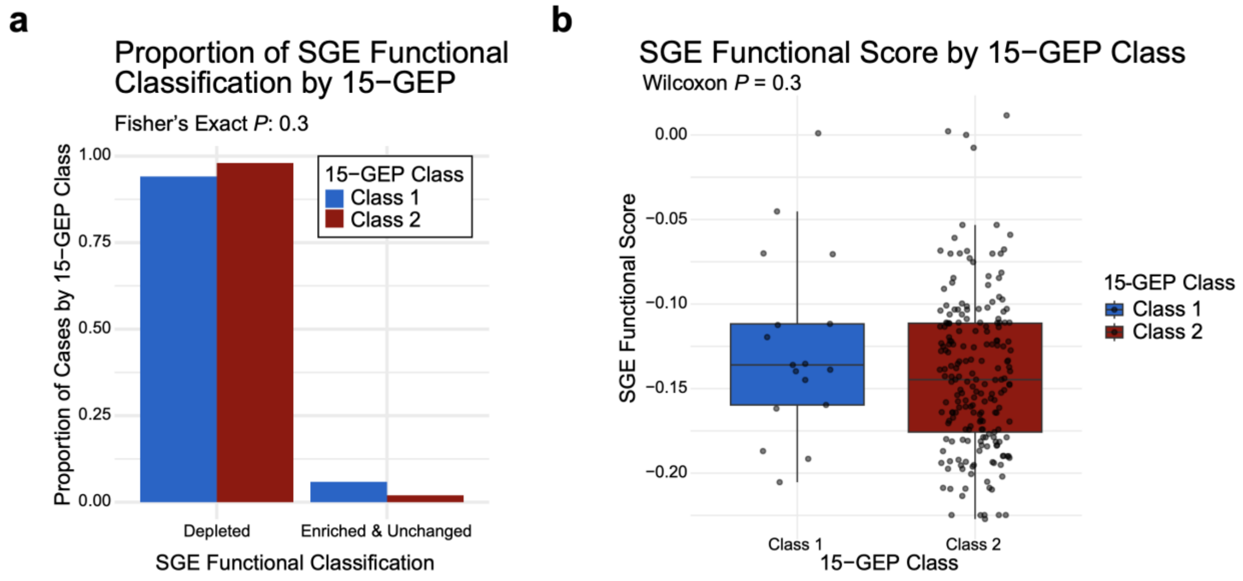

Supplementary Figure 5. Evaluation of *BAP1* Mutation Functionality by 15-GEP Class. Functional Assessment of *BAP1* Mutations (n=218 cases) with CRISPR-based Saturation Genome Editing (SGE) Database published in Water et al., 2023, with comparison of 15-GEP Class 1 (n=17 cases) versus Class 2 (n=201 cases) tumors. **a**, Bar plot depicting the frequency of deleterious functional classification (Depleted) compared to non-deleterious classification (Enriched or Unchanged) for *BAP1* mutations according to 15-GEP Class status. **b**, Box plot exhibiting the SGE functional score of *BAP1* mutations for 15-GEP Class 1 and Class 2 tumors. The box center line, lower boundary, and upper boundary shows the median, 25th percentile, and 75th percentile, respectively. The range between box boundaries indicates the interquartile range (IQR), where the lower whiskers extend to the minima, or the lowest value up to 1.5 times the IQR from the lower box boundary and the upper whiskers extend to the maxima, or the highest value up to 1.5 times the IQR from the upper box boundary. Significance for comparing functional classifications and scores was determined by two-tailed Fisher's exact test and two-tailed Wilcoxon Rank-sum test. Data available in Source Data file.

Abbreviations: 15-GEP, 15-gene expression profile; SGE, Saturation Genome Editing.

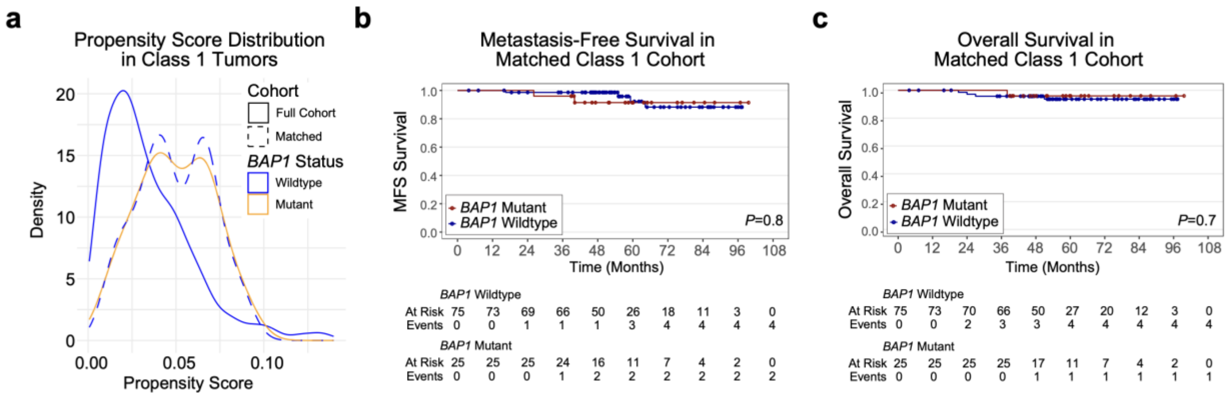

Supplementary Figure 6. Size-matched Survival Analysis of Class 1 UM by *BAP1* Mutation Status. Propensity score-matched survival analysis of Class 1 *BAP1* mutants (n=25 cases) versus Class 1 *BAP1* wildtype (n=75 cases) tumors, selected from the full cohort of Class 1 *BAP1* wildtype tumors without metastasis at baseline (n=690 cases). **a**, Distribution of propensity scores for Class 1 tumors by *BAP1* mutation status and matching status, with **b-c**, Kaplan-Meier curves for **b**, Metastasis-free survival and **c**, Overall survival analysis for the selected cohort. Relevant data provided in Source Data file.

Abbreviations: MFS, metastasis-free survival; OS, overall survival.

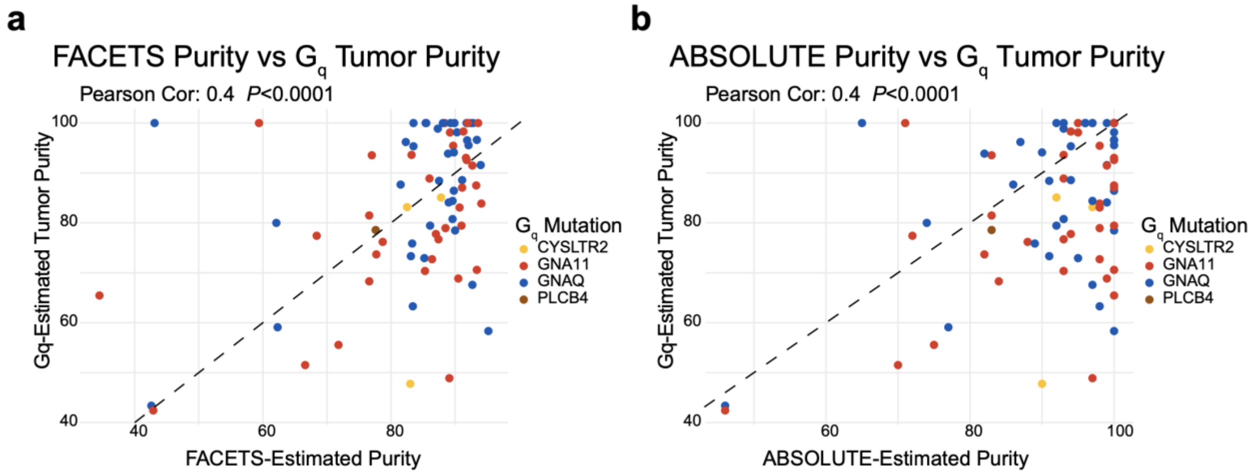

Supplementary Figure 7. Validation of tumor purity estimation by G_q mutation variant allele fraction by comparison to copy number-based methodologies reported for UM within the TCGA cohort. Correlation plots of G_q mutation-based tumor purity with previously reported purity estimates calculated with **a**, FACETS and **b**, ABSOLUTE algorithms. Plots include all UM cases with a detectable G_q mutation (n=78 cases). The dashed line indicates the identity line (y=x), while dot color indicates the representative G_q mutation. Significance determined by two-tailed t-test. Data including exact p-values provided in the Source Data file.

Abbreviations: G_q, Mutation in G_q Genes (*GNAQ*, *GNA11*, *CYSLTR2*, *PLCB4*); Pearson cor., Pearson correlation; FACETS, Fraction and Allelic Copy number Estimation from Tumor/normal Sequencing.
