# Supplementary material for: Early genetic evolution of driver mutations in uveal melanoma
Source: Nat Commun. 2025 Dec 12;16:11322. doi: 10.1038/s41467-025-66428-x (PMC12722419; doi:10.1038/s41467-025-66428-x)
Supplement: Supplementary file 2 — Description of Additional Supplementary Files [file 41467_2025_66428_MOESM2_ESM.pdf]

Description of Additional Supplementary Files

Supplementary Data 1. Table of baseline clinical annotations, patient outcomes and genetic features for all study subjects (n=1140 cases).
Abbreviations: COOG2, Collaborative Ocular Oncology Group Study 2; 15-GEP, 15-gene expression profile; SIFT, Sorting Intolerant from Tolerant algorithm; PolyPhen, Polymorphism Phenotyping algorithm; *GNAQ*, *GNA11*, *PLCB4*, *CYSLTR2*, *BAP1*, *SF3B1* and *EIF1AX* indicate the genes in which pathogenic mutations were detected; CNV, copy number variation; ng, nanograms.

Supplementary Data 2. Statistical analysis of uveal melanoma-associated mutations for all study subjects (n=1140 cases). Continuous variables were analyzed by two-tailed Wilcoxon rank-sum test. Categorical variables were analyzed by two-tailed chi-squared test or two-tailed Fisher's test if indicated by dagger (†).
Abbreviations: 15-GEP, 15-gene expression profile; No., number; mm, millimeter; *GNAQ*, *GNA11*, *PLCB4*, *CYSLTR2*, *BAP1*, *SF3B1* and *EIF1AX* indicate the genes in which pathogenic mutations were detected; BSE indicates *BAP1*, *SF3B1* and *EIF1AX*.

Supplementary Data 3. Statistical analysis of small tumors (n=131 cases) versus larger tumors (n=1009 cases). Continuous variables were analyzed by two-tailed Wilcoxon rank-sum test. Categorical variables were analyzed by two-tailed chi-squared test or two-tailed Fisher's test if indicated by dagger (†).
Abbreviations: 15-GEP, 15-gene expression profile; No., number; mm, millimeter; TP, tumor purity; *GNAQ*, *GNA11*, *PLCB4*, *CYSLTR2*, *BAP1*, *SF3B1* and *EIF1AX* indicate the genes in which pathogenic mutations were detected; BSE indicates *BAP1*, *SF3B1* and *EIF1AX*.

Supplementary Data 4. Statistical analysis of patients with BAP1-mutant tumors (n=364) versus those with BAP1-wildtype tumors (n=776). Continuous variables were analyzed by two-tailed Wilcoxon rank-sum test. Categorical variables were analyzed by two-tailed chi-squared test or two-tailed Fisher's test if indicated by dagger (†).
Abbreviations: 15-GEP, 15-gene expression profile; wt, wildtype; mut, mutant.
