# Supplementary material for: Early genetic evolution of driver mutations in uveal melanoma
Source: Nat Commun. 2025 Dec 12;16:11322. doi: 10.1038/s41467-025-66428-x (PMC12722419; doi:10.1038/s41467-025-66428-x)
Supplement: Supplementary file 4 — Reporting Summary [file 41467_2025_66428_MOESM4_ESM.pdf]

nature portfolio

Corresponding author(s): J. William Harbour

Last updated by author(s): Oct 26, 2025

Reporting Summary

Nature Portfolio wishes to improve the reproducibility of the work that we publish. This form provides structure for consistency and transparency in reporting. For further information on Nature Portfolio policies, see our Editorial Policies and the Editorial Policy Checklist.

Statistics

For all statistical analyses, confirm that the following items are present in the figure legend, table legend, main text, or Methods section.

n/a	Confirmed	
☐	☒	The exact sample size (*n*) for each experimental group/condition, given as a discrete number and unit of measurement
☐	☒	A statement on whether measurements were taken from distinct samples or whether the same sample was measured repeatedly
☐	☒	The statistical test(s) used AND whether they are one- or two-sided<br>*Only common tests should be described solely by name; describe more complex techniques in the Methods section.*
☐	☒	A description of all covariates tested
☐	☒	A description of any assumptions or corrections, such as tests of normality and adjustment for multiple comparisons
☐	☒	A full description of the statistical parameters including central tendency (e.g. means) or other basic estimates (e.g. regression coefficient) AND variation (e.g. standard deviation) or associated estimates of uncertainty (e.g. confidence intervals)
☐	☒	For null hypothesis testing, the test statistic (e.g. *F*, *t*, *r*) with confidence intervals, effect sizes, degrees of freedom and *P* value noted<br>*Give P values as exact values whenever suitable.*
☒	☐	For Bayesian analysis, information on the choice of priors and Markov chain Monte Carlo settings
☐	☒	For hierarchical and complex designs, identification of the appropriate level for tests and full reporting of outcomes
☐	☒	Estimates of effect sizes (e.g. Cohen's *d*, Pearson's *r*), indicating how they were calculated

Our web collection on statistics for biologists contains articles on many of the points above.

Software and code

Policy information about availability of computer code

Data collection

This research complies with all relevant ethical regulations. Federal Wide Assurance (FWA) from the Office of Human Research Protections (OHRP) and Institutional Review Board (IRB) or Ethics Committee approval was obtained in accordance with policies at each participating center, with oversight by the University of Miami IRB. Between January 2017 and April 2020, COOG2 enrolled 1687 subjects with UM involving the choroid, ciliary body and/or iris across 26 ocular oncology centers in the U.S. and Canada and prospectively monitored these subjects for metastatic progression and outcome. Informed written consent was obtained from each patient. Primary treatment was performed according to the standard at each center. Exclusion criteria included patient age less than 18 years, diagnosis of a uveal tumor other than UM (e.g., metastatic cancer), prior radiotherapy, inadequate sample for molecular analysis, and patient withdrawal from the study. Prior photodynamic therapy or transpupillary thermotherapy were allowed if there was evidence of tumor regrowth. No participants were excluded based on sex, ethnicity, or race. Gender was recorded from medical records and used as a proxy for biological sex in this study. No additional data on gender identity was collected. For this analysis, a data lock was performed on March 4, 2024. Subjects were not included for this report if they had a primary iris melanoma (n=101 cases), lacked adequate residual biopsy material for successful sequencing (n=212 cases) or had no detectable UMAM (n=234 cases).

REDCap (https://projectredcap.org/), a secure HIPAA compliant application48, was used for electronic data management, as previously described6. Baseline data included date of enrollment, date and method of biopsy, cytology result (if available), date and method of primary tumor treatment, patient age at study entry, sex, self-reported race and ethnicity, iris color (blue/green, intermediate, or brown), tumor diameter, tumor thickness, ciliary body involvement, and metastatic status. The American Joint Committee on Cancer (AJCC) 8th edition49 was used for tumor staging. Follow-up data included local tumor recurrence (tumor regrowth in the eye or orbit following radiotherapy or in the orbit following enucleation), metastatic status, date and location of initial metastasis, systemic status at last follow-up, and date and cause of death. Molecular test results were entered into REDCap by Castle Biosciences, which was masked to other REDCap data. Each center was masked to data entered by other centers and by Castle Biosciences. Only the coordinating center and COOG2 Data Committee had access to

all data.

Baseline and follow-up ophthalmic visits were performed as per standard of care at each center but typically included a comprehensive ophthalmic examination, fundus photography, optical coherence tomography, and ultrasonography performed at least every 3-4 months for the first year after treatment, every 4-6 months for the second year, and every 6-12 months thereafter. Baseline systemic imaging was typically performed with CT of the chest, abdomen, and pelvis. Subsequent systemic surveillance typically included imaging of the liver with CT, MRI or ultrasound at least twice a year, along with chest CT or chest x-ray at least once a year.
Only the coordinating center and COOG2 Data Committee had access to all data.

Data analysis

Tumor Sample Analysis
All subjects underwent standard clinical genetic testing of the primary tumor prior to treatment using a commercial 15-GEP prognostic test (DecisionDx®-UM, Castle Biosciences, Inc., Friendswood, TX, USA) and qPCR assay for PRAME mRNA expression (DecisionDx®-PRAME, Castle Biosciences, Inc., Friendswood, TX, USA). This testing was performed in a CAP-accredited, CLIA-certified clinical laboratory, as previously described4,43. DecisionDx®-UM employs SVM to assign each sample to Class 1 (low metastatic risk) or Class 2 (high metastatic risk), and it assigns a discriminant score as a measure of confidence based on the distance of a given sample to the SVM decision boundary32. DecisionDx®-PRAME renders a result of positive or negative based on a validated threshold {Plasseraud, 2017 #7703}.
Approximately ~25% of each clinical sample was retained for analysis on a UMAM NGS panel (DecisionDx®-UMSeq, Castle Biosciences, Inc.), as previously described28. Variants were sequenced with Ion GeneStudio S5 Prime Sequencer (ThermoFisher Scientific, Waltham, MA, USA) and processed with Ion Reporter (Version 5.6) software. Variant detection, analysis, and annotation was conducted with Ion Torrent Suite Browser (Version 5.8) and Ion Reporter using human reference sequence hg19. Sequencing quality assessment was conducted for each run, including total yield, useable reads, percent polyclonal reads, and amplicon coverage, as previously described28. Sample-specific sequencing quality metrics are included in Supplementary Data 1.
Mutations were classified as nonsense (introduction of a premature stop codon), stop-loss or start-loss (loss of stop or start codon preventing translation), frameshift insertion or deletion (shift of codon reading frame via addition or subtraction of a non-triplet set of nucleotides), non-frameshift insertion or deletion (addition or removal of a codon without shifting the reading frame), block substitution (alteration of multiple sequential codons), splice site alteration (alteration of splice donor or acceptor site), and missense (substitution of one amino acid). All of the following variants were called pathogenic: nonsense, stop-loss, start-loss, frameshift and non-frameshift insertions and deletions, and block substitutions. Splice site alterations were called pathogenic if predicted to result in splice acceptor or donor site loss or gain variant as predicted by a SpliceAI (Version 1.3) score greater than or equal to 0.5 44. Missense variants were called pathogenic if they: (1) were previously reported as pathogenic in the ClinVar Database45, (2) exhibited a SIFT (Version 5.2.2) score less than or equal to 0.05, or (3) exhibited a PolyPhen2 (Version 2.2.2) score greater than or equal to 0.5. All genetic variants that were called pathogenic were classified as tier I, II, or III according to the guidelines of the College of American Pathologists (CAP), American Society of Clinical Oncology (ASCO), and Association for Molecular Pathology (AMP)46.

Functional Assessment of BAP1 Mutations Using Saturation Genome Editing Database
BAP1 mutations involving complex alterations (≥5 nucleotide changes) were excluded from analysis and the remainder were converted from hg19 to hg38 reference genomes using the Broad Institute Liftover tool (https://liftover.broadinstitute.org/)(Version 03-03-2024). Mutations were mapped to a CRISPR-based SGE database for BAP1, matching mutations based on hg38 start position, reference allele(s), and mutant allele(s) to retrieve the previously reported SGE functional classifications and scores33. Significance of functional classification was determined by two-tailed Fisher's exact test, and significance of functional scores was determined by two-tailed Wilcoxon signed-rank test.

Calculation of Tumor Purity, Variant Allele Frequency, and Cancer Cell Fraction
Tumor purity (TP), the percentage of cells in a sample that are tumor cells, was inferred from the VAF of the Gq mutation, assuming that the Gq mutation is the founder mutation, is a heterozygous alteration, and is therefore present at 50% VAF in tumor cells. In rare cases with more than one Gq mutation, the mutation with the highest frequency (and presumably the earlier initiating mutation) was used. As such, TP=min([VAFGq-mutant x 2],100%). To validate the estimation of tumor purity based on VAF of Gq mutations, we compared tumor purity estimation using VAF of Gq mutation to that using chromosome copy number variations in the UM TCGA cohort22 using ABSOLUTE and FACETS. Statistical significance was determined using Pearson correlation (Supplementary Fig. 7).
The VAF for BAP1, SF3B1, and EIF1AX mutations was corrected for TP using the following equation: TP-corrected VAFBSE=VAFBSE ÷ TP. Samples without a detectable Gq mutation could not be corrected for VAF and, thus, were not included in analyses requiring TP-corrected VAFBSE. Next, we estimated the cancer cell fraction (CCF) for each BSE mutation, representing the proportion of UM cells that harbor a given mutation, which requires a correction for allelic copy number. SF3B1 is located on chromosome 2, which is not frequently altered in UM20,22. Thus, SF3B1 mutations were assumed to be heterozygous and CCFSF3B1=min(TP-corrected VAFSF3B1 x 2, 100%). EIF1AX is located on the X chromosome, which is also rarely lost in UM22. Thus, gender was used to calculate mutant CCFEIF1AX, where females were assumed to have an EIF1AX mutation at 50% and males at 100% of TP-corrected VAF. Thus, the CCFEIF1AX for females was calculated as CCFEIF1AX=min(TP-corrected VAFEIF1AX x 2, 100%), whereas the CCFEIF1AX for males was assumed to be equal to TP-corrected VAFEIF1AX. BAP1 is located at chromosome 3p2147, which frequently undergoes copy number loss in UM 20,22. Thus, to detect loss of heterozygosity (LOH) and calculate CCF for BAP1, we developed a custom targeted CNV sequencing panel containing 74 loci across chromosome 3p that was performed on the same sample used for the 15-GEP/PRAME classifier and UMAM NGS panel. For BAP1-mutant tumors with retention of heterozygosity for chromosome 3p, the CCFBAP1 was calculated as CCFBAP1=min(TP-corrected VAFBAP1 x 2, 100%). For tumors demonstrating LOH for chromosome 3p (LOH3p), CCFBAP1 was assumed to be equal to TP-corrected VAFBAP1.
For the custom CNV sequencing panel, B-allele frequencies and log fold-change (lfc) read depths across chromosome 3p were compared to a reference DNA panel of normals (PON), comprising peripheral blood mononuclear cell (PBMC) samples from 64 patients. Variant call format (VCF) files were analyzed using Wheeljack (https://github.com/covingto/KRCGTK/releases/tag/v0.1) (Version 0.1). Copy-number loss for chromosome 3p was detected by consistent b-allele frequencies at 100% and a decreased lfc read depth of less than 0. Isodisomy for chromosome 3p was identified by consistent b-allele frequencies at 100% and a lfc read depth of approximately 0. For downstream analyses, samples demonstrating either copy number loss or isodisomy for chromosome 3p were called as LOH3p, whereas samples without these aberrations were called as retention of heterozygosity for 3p. Calls were made by hand and adjudicated by 3 of the authors (J.J.D, C.L.D., K.R.C.). Variability across b-allele and read depth plots was used to assign confidence scores with 0, 1, 2, and 3 corresponding to very low, low, medium, and high confidence, respectively. A confidence score of 2 or 3 was required for use in downstream analyses.

For manuscripts utilizing custom algorithms or software that are central to the research but not yet described in published literature, software must be made available to editors and reviewers. We strongly encourage code deposition in a community repository (e.g. GitHub). See the Nature Portfolio guidelines for submitting code & software for further information.

Data

Policy information about availability of data

All manuscripts must include a data availability statement. This statement should provide the following information, where applicable:

- Accession codes, unique identifiers, or web links for publicly available datasets
- A description of any restrictions on data availability
- For clinical datasets or third party data, please ensure that the statement adheres to our policy

Raw sequencing data generated for this study have been deposited in the Sequence Read Archive (SRA) database and the Genotypes and Phenotypes (dbGaP) Database under accession number phs004040.v1.p1 (at http://www.ncbi.nlm.nih.gov/projects/gap/cgi-bin/study.cgi?study_id=phs004040.v1.p1). Access to the data requires an approved application through dbGaP due to patient privacy concerns. Corresponding author can be contacted and will give permission if investigator requesting the data submits reasonable research application for raw data access, an agreement for non-commercial research use only, and the requested length of time for data access. Response to request will be made within 14 days after review of request. Data will be accessible for the requested length of time proposed if request is approved.
The detailed cohort data (including mutation metrics, survival outcomes, tumor features, and patient details) analyzed in this study are available in Supplementary Information and at the Dryad Research Data Repository [https://doi.org/10.5061/dryad.z8w9ghxqk]. Forced call VCF files used for assessing BAP1 heterozygosity also deposited at the Dryad Research Data Repository. All data presented in main and supplementary figures are available in the Source Data file. For TCGA UM cohort analysis, tumor purity data was accessed from Supplementary Table provided by Robertson et al., 2018, while whole-exome sequencing analysis results were accessed from the Supplementary Data published with Field et al., 2018. BAP1 SGE functional scores and classification data from Waters et al., 2023 are available with the Supplementary Data provided by the authors and at https://github.com/team113sanger/Waters_BAP1_SGE.

Research involving human participants, their data, or biological material

Policy information about studies with human participants or human data. See also policy information about sex, gender (identity/presentation), and sexual orientation and race, ethnicity and racism.

Reporting on sex and gender	Gender was recorded based on patient self-reporting. No specific analyses were conducted to assess differences based on sex versus gender. As such, potential sex versus gender biological differences were not addressed in this research.
Reporting on race, ethnicity, or other socially relevant groupings	Race and ethnicity were recorded based on patient self-reporting. No participants were excluded on the basis of sex, ethnicity, or race. These variables were not the primary subject of this study and were not included with this study report.
Population characteristics	The age of the cohort at start of the study ranged from 18 to 99 years of age, with a median of 64.3 and a mean of 62.5. Clinical features were reported by clinicians. Treatment followed standard of care under the discretion of ocular oncologist.
Recruitment	Between January 2017 and April 2020, COOG2 enrolled 1687 subjects with UM involving the choroid, ciliary body and/or iris across 26 ocular oncology centers in the U.S. and Canada and prospectively monitored these subjects for metastatic progression and outcome. Informed consent was obtained from each patient. Primary treatment was performed according to the standard at each center. Federal Wide Assurance (FWA) from the Office of Human Research Protections (OHRP) and Institutional Review Board (IRB) or Ethics Committee approval was obtained in accordance with policies at each center. For this analysis, a data lock was performed on March 4, 2024.
Ethics oversight	This research complies with all relevant ethical regulations. Federal Wide Assurance from the Office of Human Research Protections and Institutional Review Board (IRB) or Ethics Committee approval was obtained in accordance with policies at each center. Each participating center obtained IRB approval and maintained its own IRB of record. The coordinating center's protocols numbers are 20120773 (for enrolling local participants and data collection) and 20150945 (REDCap data entry and data management for all sites).

Note that full information on the approval of the study protocol must also be provided in the manuscript.

Field-specific reporting

Please select the one below that is the best fit for your research. If you are not sure, read the appropriate sections before making your selection.

☒ Life sciences ☐ Behavioural & social sciences ☐ Ecological, evolutionary & environmental sciences

For a reference copy of the document with all sections, see nature.com/documents/nr-reporting-summary-flat.pdf

Life sciences study design

All studies must disclose on these points even when the disclosure is negative.

Sample size	This study included 1140 of the 1687 patients enrolled in COOG2 who underwent successful next-generation sequencing for canonical driver mutations.
Data exclusions	Exclusion criteria included patient age less than 18 years, diagnosis of a uveal tumor other than UM (e.g., metastatic cancer), prior radiotherapy, inadequate sample for molecular analysis, and patient withdrawal from the study. Prior photodynamic therapy or transpupillary thermotherapy were allowed if there was evidence of tumor regrowth. No participants were excluded based on sex, ethnicity, or race.

	Subjects were not included for this report if they had a primary iris melanoma (n=101), lacked adequate residual biopsy material for successful sequencing (n=212), or had no detectable UMAM (n=234).
Replication	Replication was not conducted with this study due to the limited genetic material obtainable by fine needle biopsy and patient safety issues that would arise from multiple biopsies that are not clinically indicated.
Randomization	No randomization was done nor indicated for this type of non-interventional study.
Blinding	All centers were blinded to data from other centers. Castle Bioscience entered clinical molecular testing data via REDCap but was blinded to patient data for all centers.

Reporting for specific materials, systems and methods

We require information from authors about some types of materials, experimental systems and methods used in many studies. Here, indicate whether each material, system or method listed is relevant to your study. If you are not sure if a list item applies to your research, read the appropriate section before selecting a response.

Materials & experimental systems

n/a	Involved in the study
☒	☐ Antibodies
☒	☐ Eukaryotic cell lines
☒	☐ Palaeontology and archaeology
☒	☐ Animals and other organisms
☐	☒ Clinical data
☒	☐ Dual use research of concern
☒	☐ Plants

Methods

n/a	Involved in the study
☒	☐ ChIP-seq
☒	☐ Flow cytometry
☒	☐ MRI-based neuroimaging

Clinical data

Policy information about clinical studies

All manuscripts should comply with the ICMJE guidelines for publication of clinical research and a completed CONSORT checklist must be included with all submissions.

Clinical trial registration	Not applicable
Study protocol	Not applicable
Data collection	Between January 2017 and April 2020, COOG2 enrolled 1687 subjects with UM involving the choroid, ciliary body and/or iris across 26 ocular oncology centers in the U.S. and Canada and prospectively monitored these subjects for metastatic progression and outcome. Quality next-generation sequencing data and subsequent analysis was conducted on 1140 cases after exclusion criteria.
Outcomes	Overall Survival, Metastasis-Free Survival

Plants

Seed stocks	*Report on the source of all seed stocks or other plant material used. If applicable, state the seed stock centre and catalogue number. If plant specimens were collected from the field, describe the collection location, date and sampling procedures.*
Novel plant genotypes	*Describe the methods by which all novel plant genotypes were produced. This includes those generated by transgenic approaches, gene editing, chemical/radiation-based mutagenesis and hybridization. For transgenic lines, describe the transformation method, the number of independent lines analyzed and the generation upon which experiments were performed. For gene-edited lines, describe the editor used, the endogenous sequence targeted for editing, the targeting guide RNA sequence (if applicable) and how the editor was applied.*
Authentication	*Describe any authentication procedures for each seed stock used or novel genotype generated. Describe any experiments used to assess the effect of a mutation and, where applicable, how potential secondary effects (e.g. second site T-DNA insertions, mosiacism, off-target gene editing) were examined.*
